# Lithocholic Acid Alleviates Deoxynivalenol-Induced Lethal Cholesterol Metabolic Abnormalities in IPI-2I Cells

**DOI:** 10.3390/metabo12070659

**Published:** 2022-07-17

**Authors:** Yanwei Li, Fang Gu, Haotian Gu, Ping Hu, Hui-Xin Liu, Demin Cai

**Affiliations:** 1 College of Animal Science and Technology, Yangzhou University, Yangzhou 225009, China; lyw080805@163.com (Y.L.); fanggu1011@163.com (F.G.); guhaotian1998@163.com (H.G.); pinghu@yzu.edu.cn (P.H.); 2 Health Sciences Institute, China Medical University, Shenyang 110122, China; 3 Liaoning Key Laboratory of Obesity and Glucose/Lipid Associated Metabolic Diseases, China Medical University, Shenyang 110122, China

**Keywords:** lithocholic acid, deoxynivalenol, cholesterol, apoptosis, bile acid

## Abstract

Deoxynivalenol (DON) is a secondary metabolite of fungi. Ingestion of feed containing DON causes severe intestinal damage in humans and animals, possibly due to cholesterol-enriched lipid raft abnormalities. Cholic acid (CA) and lithocholic acid (LCA) are metabolites of cholesterol transformation, which have been proven to benefit epithelial cell proliferation and reduce intestinal inflammation and lesions. Therefore, we aimed to study the protective roles of CA and LCA administration on the DON-exposed intestinal epithelial cells (IPI-2I) and the underlying mechanisms involved in cholesterol metabolism. We found that LCA pretreatment, but not CA, alleviated the reduction of cell numbers caused by DON exposure. Furthermore, we demonstrate that LCA restored the DON-induced cell apoptosis by reducing the cleaved caspase 3 and cleaved PARP-1 expression. DON-increased cellular cholesterol and bile acid contents were significantly reduced when LCA was co-treated. Further transcriptomic analysis revealed that the aberrant cholesterol homeostasis genes profile was observed in the cells exposed to DON or pretreated with LCA. We also validated that the key genes involved in cholesterol biosynthesis and transformation (cholesterol to bile acids) were strongly inhibited by the LCA treatment in the DON-exposed cells. Together, this study demonstrated that LCA ameliorated DON-caused toxic apoptosis in IPI-2I cells by maintaining cholesterol metabolism. We suggest that as an endogenous metabolite, LCA may be used as a therapeutic and/or integrated into a dietary intervention against mycotoxin toxicity.

## 1. Introduction

The secondary metabolites produced by fungi, i.e., mycotoxins, exist in the production, processing, and storage of agricultural products (mainly cereals) [1,2]. Ingestion of mycotoxin leads to its accumulation in organs and tissues of humans and animals, even a low dose of mycotoxin can cause severe damage and poisoning [3,4]. Mycotoxins and their derivatives are closely linked to food safety, in which deoxynivalenol (DON) is one of the most widespread ones [5]. DON is a mycotoxin present in grains infected with *Fusarium graminearum* and *F. culmorum*. It mainly exists in wheat, barley, rye, and corn [6]. Chronic low-dose exposure to DON contamination triggers vomiting, diarrhea, food rejection, weight loss, and even death in animals [7,8,9,10]. DON exposure causes huge economic losses in the livestock sector and imposes threats on food safety for humans. However, till today it is still difficult to eliminate DON from the environment, due to its strong stability and wide range of existence. Therefore, it is urgent to unravel the regulatory mechanisms of DON toxicology in the endogenous pathway of animals.

One major function of intestinal epithelial cells is to act as a natural barrier to prevent the penetration of toxins and pathogens in the intestine. Indeed, the gastrointestinal tract (GI) is the first line of defense against DON exposure [11]. Pigs are highly susceptible to DON exposure. It has been reported that DON ingestion breaks down the intestinal barrier integrity by disrupting the expression of key genes involved in tight junction regulation in pigs [12,13]. Furthermore, Wang et al. reveal that DON impaired the intestinal barrier integrity in weaned piglets due to caspase-12 activation [14]. In vitro studies have shown that at the cellular level, DON induces apoptosis, promotes inflammatory responses, and reduces the expression of tight junction protein claudin-4 in intestinal epithelial cells [15,16,17]. Given the importance of bile acids for maintaining intestinal tight junction structure and barrier function [18], a question raised is whether bile acids are critical for the protection from DON exposure by improving intestinal guarding efficiency. 

Bile acids, in particular, the primary ones, are transformed from cholesterol metabolism by cholesterol 7α-hydroxylase (CYP7A1) and sterol 27-hydroxylase (CYP27A1) in mammals [19]. CYP27A1-driven generation of 27-hydroxycholesterol is further hydroxylated by oxysterol 7α-hydroxylase (CYP7B1). Afterward, the primary bile acids cholic acid (CA) and chenodeoxycholic acid (CDCA) are secreted into the duodenum. Most bile acids (95%) are then reabsorbed in the ileum, while unabsorbed bile acids enter the colon and are metabolized into secondary bile acids through dehydroxylation [20]. Only a few known bacteria (e.g., *Lachnospiraceae* and *Ruminococcaceae* families) perform 7α dehydroxylation to produce lithocholic acid (LCA) [21]. LCA is one of the most abundant secondary bile acids, with concentrations of about 160 μM in human cecal contents [22]. Importantly, LCA has been demonstrated to promote the regeneration of intestinal epithelium by activating G-protein-coupled bile acid receptor TGR5 in intestinal stem cells against epithelium damage and the subsequent barrier destruction [10]. Additionally, LCA plays a protective role in the dextran sulfate sodium (DSS) colitis model, and intragastric administration of LCA performs an obvious repairing of intestinal barrier integrity [23]. 

In this study, we therefore investigated the possible anti-mycotoxin actions of two candidate bile acids, CA and LCA, in the DON-exposed porcine IPI-2I cells focusing on cell survival capacity. We detected the cholesterol and bile acid contents accumulation in the DON-exposed cells, together with the protein expressions of the key enzymes in the processes of cholesterol biosynthesis, efflux, and cholesterol transformation to bile acids with or without LCA treatment. A transcriptomic pattern provided the signature genes of cholesterol metabolism, as a potential mechanism behind LCA-restored toxicity caused by DON. 

## 2. Results 

### 2.1. LCA Alleviates DON-Induced IPI-2I Cell Death 

**Figure 1:**
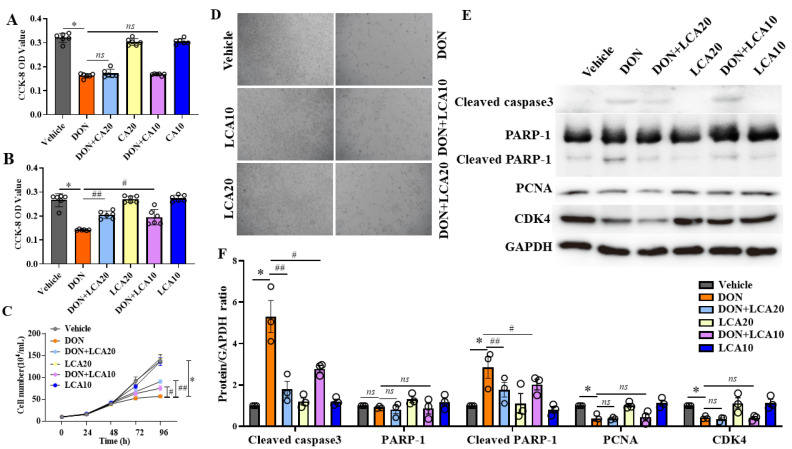
LCA pretreatment alleviates the DON-induced IPI-2I cell death. With CA (**A**) or LCA (**B**), pretreatment in IPI-2I cells for 24 h, DON was added for another 48 h to process CCK-8 analysis. (**C**) Cell number measurement at different time points (0, 24, 48, 72, 96 h) with indicated treatments. (**D**) LCA pretreatment for 24 h, followed by DON for another 48 h, and cell morphology was captured at 40× magnification. (**E**) Western blotting analyses of cleaved caspase 3, PARP-1, PCNA, CDK4, and GAPDH (used as an internal reference). (**F**) The relative expression levels of cleaved caspase 3, PARP-1, cleaved PARP-1, PCNA, and CDK4 were determined by GAPDH standardization. Results are mean ± SD. *n* = 3. * represents DON group vs. vehicle group *p* < 0.05, # represents DON + LCA10 group vs. DON group *p* < 0.05. ## represents DON + LCA20 group vs. DON group *p* < 0.05. ns represents no significant difference between the two groups.

To test the protective effects of CA and LCA on intestinal epithelial cells exposed to DON, we first used CA and LCA to pretreat IPI-2I cells for 24 h and then treated the cells with DON for 48 h. The CCK-8 measurement of cell viability showed that LCA pretreatment alleviated the reduction of IPI-2I cell number induced by DON, but not CA (Figure 1A,B). Therefore, we focused on the protective reactions of LCA in the following tests. In association with the CCK-8 results, LCA also recovered the cell number of IPI-2I cells using cell counting analysis (Figure 1C,D). To further investigate how LCA alleviated DON-reduced cell number of IPI-2I, the key proteins involved in cell proliferation and apoptosis including cell cyclin kinase (CDK4), DNA polymerase helper protein (PCNA), PARP-1, and cleaved caspase 3 were measured. Indeed, DON induced the expression of the apoptotic proteins (cleaved PARP-1, cleaved caspase 3), and inhibited the expression of CDK4 and PCNA significantly. Whereas LCA decreased the expression of DON-induced apoptotic proteins (Figure 1E,F). These results suggest that LCA attenuates DON-induced IPI-2I cell death probably via the apoptotic pathway. 

### 2.2. LCA Restores the Cholesterol Homeostasis Pathway Altered in DON-Treated IPI-2I Cells 

**Figure 2:**
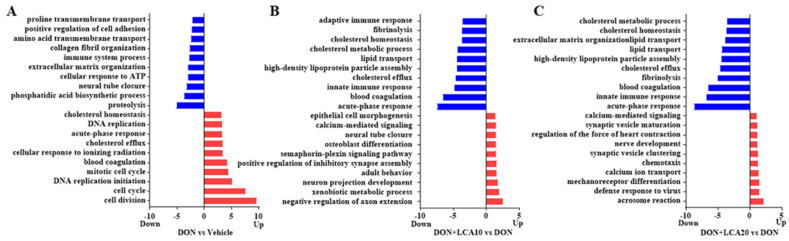
LCA alleviates the dysregulation of cholesterol homeostasis induced by DON. The genes involved in the cholesterol homeostasis pathway and cholesterol efflux pathway were the most abundant using GO analysis. (**A**) DON versus vehicle; (**B**) DON + LCA10 versus DON; (**C**) DON + LCA20 versus DON. Red and blue represent the upregulated or downregulated signaling pathways when comparing DON versus vehicle, DON + LCA10 versus DON, or DON + LCA20 versus DON, respectively.

To uncover the critical pathway of LCA control in DON-treated IPI-2I cells, we performed an RNA-seq analysis using the samples from DON, LCA10, LCA20, DON + LCA10, DON + LCA20, and the vehicle group. The gene ontology (GO) analysis reveals that the cholesterol homeostasis and the cholesterol efflux pathways were strongly activated when IPI-2I cells were treated with DON (Figure 2A). Interestingly, the different concentrations of LCA (10, 20 μmol/L) pretreatment showed a similar alteration of these two pathways (Figure 2B,C). These results suggest that cholesterol homeostasis may be the predominant event in the IPI-2I cells in response to LCA and/or DON. 

### 2.3. LCA Modulates Cellular Cholesterol Content Homeostasis 

**Figure 3:**
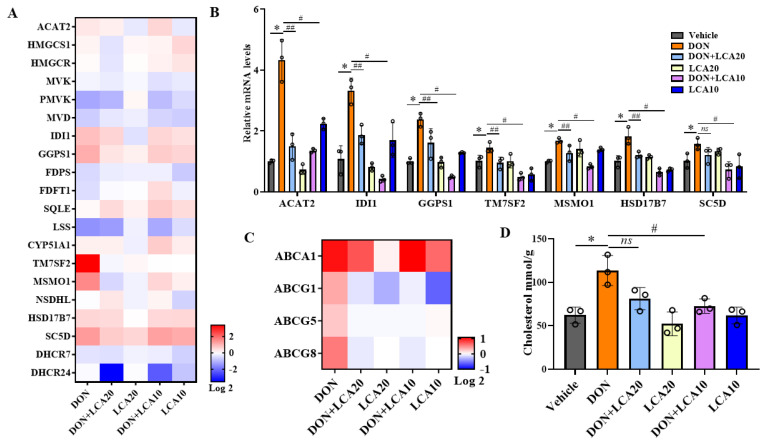
LCA modulates cellular cholesterol content homeostasis. (**A**) Heatmap analysis of gene expression (RNA-seq) of key enzymes in cholesterol *de novo* synthesis (log2 transformed, normalized to vehicle). (**B**) qRT-PCR detects the gene expression of key enzymes in cholesterol synthesis, the relative expression levels were evaluated by qRT-PCR. (**C**) Heatmap analysis of gene expression (RNA-seq) in cholesterol efflux. (**D**) The level of total cholesterol in cells under different treatments was standardized by protein concentration. Results are mean ± SD. *n* = 3. * represents DON group vs. vehicle group *p* < 0.05. # represents DON + LCA10 group vs. DON group *p* < 0.05. ## represents DON + LCA20 group vs. DON group *p* < 0.05. ns represents no significant difference between the two groups.

Consistent with the GO results, our pathway-focused analysis demonstrated that the vast majority of the cholesterol-biosynthesis genes were significantly upregulated by the DON treatment, including *IDI1*, *GGPS1*, *TM7SF2*, *MSMO1*, *HSD17B7,* and *SC5D.* We also found that LCA could decrease these genes against DON exposure (Figure 3A). We further verified the RNA-seq results by RT-qPCR (Figure 3B). The genes *ABCG1*, *ABCG5,* and *ABCG8* involved in cholesterol efflux were significantly downregulated at the transcriptional level in the LCA-treated cells exposed to DON, compared to that of DON treatment alone (Figure 3C). The alteration of the gene profiles is an indicator of changed cellular cholesterol content in response to single DON or combined DON and LCA treatment. We thus measured the total cholesterol concentration in IPI-2I cells. As expected, DON triggered a hyper-cholesterol accumulation in cells while LCA efficiently reduced the overt cholesterol content (Figure 3D). These results suggested that the cholesterol content change may be associated with cell death responses in IPI-2I cells with the respective treatments. 

### 2.4. LCA Reduces DON-Increased Bile Acids Levels in IPI-2I Cells

**Figure 4:**
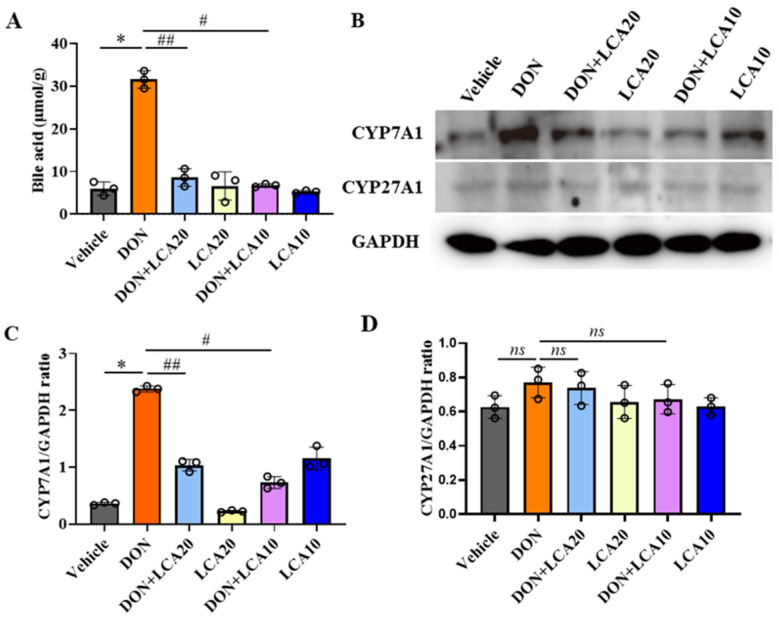
LCA reduces DON-increased bile acids in IPI-2I cells. (**A**) Bile acid levels measurement in IPI-2I with indicated treatments. (**B**) Western blot detected the expression of CYP7A1, CYP27A1, and GAPDH protein. (**C**) The relative expression levels of CYP7A1 were determined by GAPDH standardization. (**D**) The relative expression levels of CYP27A1 were determined by GAPDH standardization. Results are mean ± SD. *n* = 3. * represents DON group vs. vehicle group *p* < 0.05. # represents DON + LCA10 group vs. DON group *p* < 0.05. ## represents DON + LCA20 group vs. DON group *p* < 0.05. ns represents no significant difference between the two groups.

Given that cholesterol homeostasis is maintained by several pathways such as cholesterol biosynthesis, uptake, efflux, cholesterol transformation, etc., we next found that the bile acid content was dramatically increased, along with the higher concentrations of cholesterol induced by the DON treatment in IPI-2I cells. Again, LCA significantly reduced the DON-triggered bile acids production with both doses of 10 and 20 μmol/L (Figure 4A). Elevated levels of bile acids are often attributed to the activated process of converting cholesterol to bile acids. Thus, we studied the expression of key rate-limiting enzymes CYP7A1 and CYP27A1 in this transformation. The results showed that DON significantly increased the protein expression of CYP7A1, while LCA pretreatment obviously caused a reduction of its expression (Figure 4B,C). In contrast, DON did not affect the expression of CYP27A1 (Figure 4B,D). These data suggested that LCA reduced DON-caused bile acids increment in IPI-2I cells, possibly via the activation of CYP7A1.

### 2.5. LCA Reprograms DON-Caused Abnormal Transcripts of Cholesterol Transformation

**Figure 5:**
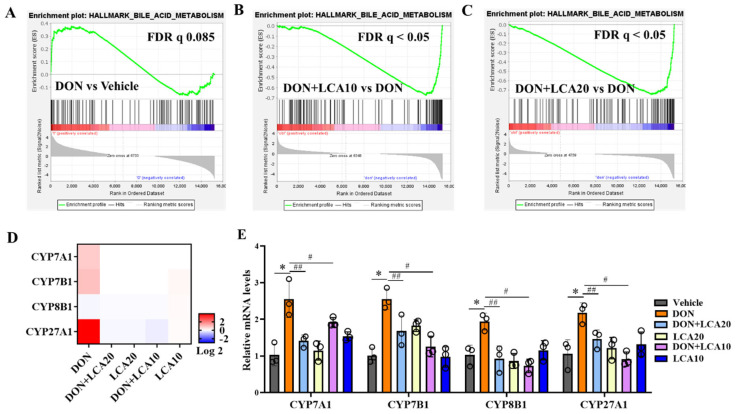
LCA reprograms DON-caused abnormal transcripts of cholesterol transformation. (**A**–**C**) GSEA analysis of metabolic pathway of cholesterol transformation with indicated treatments. (**D**) Gene transcripts profile of transcriptome analysis. (**E**) qRT-PCR analysis of the gene expression of CYP7A1, CYP7B1, CYP8B1, and CYP27A1. FDR, false–discovery rate. Results are mean ± SD. *n* = 3. * represents DON group vs. vehicle group *p* < 0.05. # represents DON + LCA10 group vs. DON group *p* < 0.05. ## represents DON + LCA20 group vs. DON group *p* < 0.05.

We demonstrated that the key enzymes of cholesterol transformation were vulnerable to DON or LCA treatment. Next, we performed a GSEA analysis using the RNA-seq data and demonstrated that the increased cholesterol transformation gene pathway caused by DON was remarkably rescued when the IPI-2I cells were pretreated with LCA compared to that of the group without LCA (Figure 5A–C). The pathway-focused transcriptome results of the critical genes including CYP7A1, CYP7B1, CYP8B1, and CYP27A1 were significantly elevated in DON-treated cells, and dramatically downregulated by LCA administration (Figure 5D). Moreover, the altered transcriptional profile was also validated by qRT-PCR, and further assured the effect of LCA on the cholesterol transformation pathway to resist DON exposure (Figure 5E).

## 3. Discussion

Deoxynivalenol contaminates cereal-based foods that are used to feed humans and animals, increasing the risks of diseases, organ injury, and even death [24,25,26]. It is well-known that the gastrointestinal tract is the vital physical barrier against mycotoxin entry and acts as the first line of defense [27]. Herein, we observed significant cell apoptosis reflected by caspase enzyme activities and PARP protein expression in the porcine intestinal epithelial cells exposed to DON treatment with different concentrations. Given the important roles of bile acids in the gut protection in vivo [18], we tried to demonstrate the recovery of DON-induced IPI-2I cell death. The primary bile acid CA, in different doses in the DON-exposed cells, has no obvious effects on the cell number and growth compared to that of a single DON treatment. In contrast, the secondary bile acid, LCA, showed a strong anti-mycotoxin capacity. Notably, in the previous studies on cancer cells, LCA was more likely to inhibit cell viability and served as an anti-cancer agent [28,29]. However, in the normal cell lines, we would like to address the protection role to suppress cell apoptosis. Indeed, the specific roles in cell growth and death of LCA in different cell lines has been demonstrated [30]. Herein, the reduced cleavage of caspase 3 and RARP-1 enrolled in apoptosis were observed in the cell with the treatment of LCA co-treated with DON compared to that of DON treatment alone. 

Accumulating evidence has shown that lipids, especially those that constitute the rafts of cellular membrane, play protective roles against the exposure of mycotoxins such as OTA and fumonisin B1, possibly via maintaining membrane properties and H^+^-ATPase activity [31,32]. This would lead to the repair of the mycotoxin-induced structural failure of the cell membrane by activating ceramide synthesis at the endoplasmic reticulum [33,34]. Given that those plasma membrane microdomains are enriched in cholesterol and sphingolipids, we evaluated the cholesterol metabolism in response to DON and/or LCA treatment in IPI-2I cells. DON increased the cholesterol concentration in IPI-2I cells. This suggests that cholesterol enrichment in lipid rafts may facilitate DON entry and thus promote cell death. Moreover, a high concentration of cholesterol accumulated the total bile acids production. It is noted that LCA, a secondary bile acid produced only by microbiota, is synthesized in the colon and thereafter transferred to blood circulation [20,35]. The increased CA in the cells in vitro would not remarkably affect LCA concentration. Therefore, DON-triggered CA accumulation showed no response to additional CA supply, but high sensitization to LCA. 

Consistent with the increased cellular contents of cholesterol and bile acids, the cholesterol metabolism program is highly enriched in IPI-2I cells in response to DON. Again, the selected genes of key enzymes involved in cholesterol metabolism were upregulated in DON-exposed cells. The different appearance in the liver features an incongruity between mRNA and protein expression in a negative-feedback loop [36,37]. In the IPI-2I cell, an aberrant cholesterol metabolism is a lethal event in response to DON exposure, which cannot resemble the compensation of cholesterol and bile acids in the liver. Interestingly, the rate-limiting enzyme CYP7A1 involved in cholesterol–bile acids transformation was significantly increased in IPI-2I cells in association with elevated total bile acids content with treatment. It is worth mentioning that CA biosynthesis has mainly occurred in the liver as documented and we provide a new potential reaction site of the CA synthesis in the present study. Importantly, whether the high expression of CYP7A1 implies other vital functions of this enzyme in the gut is unknown and should be explored in the near future. 

It has been reported that LCA modulates cholesterol *de novo* biosynthesis using a labeled [3H] cholesterol assay in vivo despite the unclear mechanisms [38]. In line with this finding, we revealed the downregulated cholesterol biosynthesis genes possibly induced by LCA administration in DON-exposed IPI-2I cells. Scientific reasons for this LCA action are closely linked to a critical play of nuclear receptor RORγ [39]. RORγ is the dominant transcription factor over the classic cholesterol metabolic modulator SREBP2 to positively drive cholesterol biosynthesis, especially in the scenario of mycotoxin exposure. Interestingly, recent studies have provided some clues that a derivative of LCA [40], 3-oxoLCA, is defined to be a novel inhibitor of RORγ, suggesting that the LCA reduced cholesterol *de novo* synthesis in DON-exposed IPI-2I cells in the current study, probably due to RORγ inactivity. As mentioned before, given that LCA was predominantly derived from intestinal bacteria, or the 3-oxoLCA as newly discovered [41,42], our follow-up study would validate the deep mechanisms of RORγ-mediated protection by LCA against mycotoxin exposure.

In summary, the present study demonstrates that the DON toxicity on cell growth and proliferation in intestinal epithelial cells could be recused by a secondary bile acid. The cholesterol metabolic abnormality may be the critical event to link the association between LCA and mycotoxin interaction and regulation. Due to the similarities to humans in anatomy, body size, physiology, metabolism, and omnivorous habits, pigs are better suited biomedical models for studying the mechanisms of mycotoxin-induced toxic responses compared to other animal models [43]. Although mostly descriptive, the results presented here provide the first evidence that LCA is a potent candidate for anti-mycotoxin therapeutics and related dietary interventions in humans and animals. 

## 4. Materials and Methods

### 4.1. Cell Culture

Porcine ileum epithelial cell line IPI-2I (purchased from European Collection of Authenticated Cell Cultures (ECACC)) were cultured in RPMI-1640 (Hyclone, Logan, UT, USA) supplemented with 10% fetal bovine serum (Hyclone, USA), 100 U/mL penicillin (Solarbio, Beijing, China), and 100 μg/mL streptomycin (Solarbio, China) at 37 °C with 5% CO_2_ in a humidified incubator.

### 4.2. Cell Counting Kit-8 (CCK-8) Assay

When the cells in the culture dish grew to 90%, the cells were digested with trypsin and the number was calculated under the microscope. In total, 3 × 10^3^ cells were seeded into a 96-well plate. When these cells grew to 50%, LCA (10, 20 μmol/L), CA (10, 20 μmol/L) or DMSO was added for 24 h. There, cells were further exposed to DON (250 ng/mL) or DMSO for another 48 h. Next, 10 μL CCK-8 (Biosharp, Hefei, China) and 100 μL incomplete medium were added to each well with a 1 h incubation at 37 °C. In the microplate reader, the wavelength of 450 nm was selected to detect the absorbance. 

### 4.3. Cell Counting Experiment

The cells in the 6-well plate were divided into vehicle, DON (250 ng/mL), LCA (10 μmol/L), LCA (20 μmol/L), DON + LCA (10 μmol/L), and DON + LCA (20 μmol/L) group. LCA (Shanghai yuanye, Shanghai, China) at concentrations of 10 or 20 μmol/L was added to the indicated wells for 24 h. Then, DON (J&K Scientific, Beijing, China) or DMSO were added accordingly for another 48h. The number of cells was counted at 0, 24, 48, 72, and 96 h under the microscope.

### 4.4. qRT-PCR and RNA-seq

The cells in the 6-well plate were harvested and lysed using 1 mL trizol (Invitrogen, Waltham, MA, USA) to extract total RNA and to be reverse-transcribed into cDNA according to the instructions (Vazyme, Nanjing, China), and the cDNA was subjected to qRT-PCR (Vazyme, Nanjing, China). The primers are shown in Table 1. The relative expression levels of genes were analyzed using the 2^−ΔΔCT^ method. The RNA-seq libraries were generated as previously described [36,37] with modifications. Total RNA (2 μg) was prepared using Illumina Tru-Seq RNA Sample Prep Kit according to the manufacturer’s instructions. The quality of RNA-seq libraries was tested with an Agilent Bioanalyzer (Agilent Technologies). The high throughput sequencing was performed on a BGISEQ-2000 at BGI Tech. (Wuhan, China). The sequence data in FASTQ format was analyzed using the standard BWA–Bowtie–Cufflinks workflow. Sequence reads were mapped to susScr3 assembly with BWA and Bowtie software. The cufflinks package was used for transcripts assembly, quantification of normalized gene and isoform expression, and analysis of differentially expressed genes. The quantitative analysis of FPKM was selected for subsequent data analysis. GSEA software was used to analyze the differential signal pathway, and Gene Ontology (GO) analysis was performed using DAVID Bioinformatics Resources with gene expression differences greater than 2. 

### 4.5. Western Blot Assay

The cells were pretreated with LCA for 24 h, DON was added, and treated for 48 h, the medium was discarded and washed with PBS. Then, 200 μL RIPA lysis buffer was added to lyse the cells, extract the total protein of the cells, and determine the protein concentration with a BCA kit (Phygene, Fuzhou, China). A total of 30 μg protein sample was added to 10% SDS-PAGE separation gel, the separated protein was transferred to PVDF membrane, and 5% skimmed milk powder sealed PVDF membrane, and then the primary antibody was incubated on a 4 °C shaker overnight. The primary antibodies were PARP-1 (Cell signaling technology, Danvers, MA, USA), Cleaved caspase 3 (Cell signaling technology, USA) protein, CDK4 (Proteintech, Wuhan, China), and PCNA (Proteintech, Wuhan, China), respectively. The PVDF membrane was washed with TBST solution on the room temperature shaking table and this was repeated 3 times for 10 min each time. The secondary antibody was incubated in the ratio of 1:3000 and on the shaking table for 2 h. The PVDF membrane was washed again and then developed with ECL luminescent solution. Western blot detected the expression of GAPDH and used GAPDH as the internal reference. 

### 4.6. Detection of Cholesterol and Bile Acids 

When the cells were pretreated with LCA for 24 h, DON was added and treated for 48 h, the RPMI-1640 medium was discarded and washed with PBS, 200 μL PBS was added to 6-well plates, and the cells were scraped off with a cell brush. The cell suspension was transferred to a centrifuge tube and centrifuged at 2000 rpm for 10 min. The supernatant of centrifugation was used to determine the protein concentration, cholesterol concentration, and bile acid concentration in the supernatant. Cholesterol was measured with a biochemical analyzer, bile acids were measured with a total bile acid assay kit (Nanjing jiancheng bioengineering institute, Nanjing, China), and normalized to total protein.

### 4.7. Statistical Analysis

Graphpad prism 8.0 software was selected for data analysis, the data are expressed as the mean ± SD of at least three independent experiments. *p* < 0.05 was considered significant.

## Figures and Tables

**Table 1:** The table of gene primer sequences.

Gene	Sequence
CYP7A1 Forward CYP7A1 Reverse	TAGCAGGCTTCCCGATTC CTGACCAGTTCCGAGATGTG
CYP27A1 Forward CYP27A1 Reverse	TGTGGCTCGCATCGTTC TCACCTGGCAGCTCCTT
CYP8B1 Forward CYP8B1 Reverse	GCAGGCAAGAAGATCCACCACTAC TGACCATGAGCAGCACAAAGAGC
CYP7B1 Forward	ACATCATTTAGGCTTGCTC
CYP7B1 Reverse	TGTAGTGTCAGGTCCTCTTG
ACAT2 Forward ACAT2 Reverse	TAATGATGGTGCTGCTGCTGTGG GCTTGCTTTATTGCCGGGATTGG
IDI1 Forward IDI1 Reverse	TGCTCCAACAACGATCAGATGCC TTAAACGCCTCTGTGCTGCTCTTC
GGPS1 Forward GGPS1 Reverse	GTGGCACACAGCATCTATGGAA GCCTTGGCCCTGATGTAGTT
TM7SF2 Forward TM7SF2 Reverse	ACCCACGCATCTGTTCCTTTGAC GGAAGCCATTGACCAGCCACATG
MSMO1 Forward MSMO1 Reverse	CCTGGGTGACCGTTCGTTTGATAG GGTGGAAGTCATGGTGACGAGAAC
HSD17B7 Forward HSD17B7 Reverse	GAGGACATCCAGCACAGCAAAGG CGGTTCAAAGCCACACTCACAAAG
SC5D Forward SC5D Reverse	ACCCTCTGGATGGCTTCCTTCAG AGCTCTGGGGAACACGGAAGTC
GAPDH Forward GAPDH Reverse	GATTCCACCCACGGCAAGTTCC AGCACCAGCATCACCCCATTTG

## Data Availability

The datasets used and analyzed during the current study are available from the corresponding author on reasonable request. The data are not publicly available due to restrictions on privacy.

## References

[1] Zhao S., Zhang J., Sun X., Yangzom C., Shang P. (2022). Mitochondrial calcium uniporter involved in foodborne mycotoxin-induced hepatotoxicity. Ecotoxicol. Environ. Saf..

[2] Ganesan A.R., Mohan K., Karthick Rajan D., Pillay A.A., Palanisami T., Sathishkumar P., Conterno L. (2022). Distribution, toxicity, interactive effects, and detection of ochratoxin and deoxynivalenol in food: A review. Food Chem..

[3] Tkaczyk A., Jedziniak P. (2021). Mycotoxin biomarkers in pigs-current state of knowledge and analytics. Toxins.

[4] Liew W.P., Mohd-Redzwan S. (2018). Mycotoxin: Its impact on gut health and microbiota. Front. Cell. Infect. Microbiol..

[5] Streit E., Naehrer K., Rodrigues I., Schatzmayr G. (2013). Mycotoxin occurrence in feed and feed raw materials worldwide: Long-term analysis with special focus on Europe and Asia. J. Sci. Food Agric..

[6] Guo H., Ji J., Wang J.S., Sun X. (2020). Deoxynivalenol: Masked forms, fate during food processing, and potential biological remedies. Compr. Rev. Food Sci. Food Saf..

[7] Thapa A., Horgan K.A., White B., Walls D. (2021). Deoxynivalenol and zearalenone-synergistic or antagonistic agri-food chain co-contaminants?. Toxins.

[8] Hooft J.M., Bureau D.P. (2021). Deoxynivalenol: Mechanisms of action and its effects on various terrestrial and aquatic species. Food Chem. Toxicol..

[9] Loibl P., Windisch W., Preissinger W. (2020). Examination of high-resolution feed intake data of growing-finishing pigs confronted with high deoxynivalenol contents present in their feed. Czech J. Anim. Sci..

[10] Sorrentino G., Perino A., Yildiz E., El Alam G., Sleiman M.B., Gioiello A., Pellicciari R., Schoonjans K. (2020). Bile Acids Signal via TGR5 to activate intestinal stem cells and epithelial regeneration. Gastroenterology.

[11] Ward J.B.J., Lajczak N.K., Kelly O.B., O’Dwyer A.M., Giddam A.K., Ni Gabhann J., Franco P., Tambuwala M.M., Jefferies C.A., Keely S. (2017). Ursodeoxycholic acid and lithocholic acid exert anti-inflammatory actions in the colon. Am. J. Physiol. Gastrointest Liver Physiol..

[12] Akbari P., Braber S., Gremmels H., Koelink P.J., Verheijden K.A.T., Garssen J., Fink-Gremmels J. (2014). Deoxynivalenol: A trigger for intestinal integrity breakdown. Faseb. J..

[13] Savard C., Provost C., Alvarez F., Pinilla V., Music N., Jacques M., Gagnon C.A., Chorfi Y. (2015). Effect of deoxynivalenol (DON) mycotoxin on in vivo and in vitro porcine circovirus type 2 infections. Vet. Microbiol..

[14] Wang S., Yang J., Zhang B., Wu K., Yang A., Li C., Zhang J., Zhang C., Rajput S.A., Zhang N. (2018). Deoxynivalenol impairs porcine intestinal host defense peptide expression in weaned piglets and IPEC-J2 Cells. Toxins.

[15] Lan M., Han J., Pan M.H., Wan X., Pan Z.N., Sun S.C. (2018). Melatonin protects against defects induced by deoxynivalenol during mouse oocyte maturation. J. Pineal. Res..

[16] Pinton P., Braicu C., Nougayrede J.P., Laffitte J., Taranu I., Oswald I.P. (2010). Deoxynivalenol impairs porcine intestinal barrier function and decreases the protein expression of claudin-4 through a mitogen-acitivated protein kinase-dependent mechanism. J. Nutr..

[17] Kang R., Li R., Dai P., Li Z., Li Y., Li C. (2019). Deoxynivalenol induced apoptosis and inflammation of IPEC-J2 cells by promoting ROS production. Environ. Pollut..

[18] Ghosh S., Whitley C.S., Haribabu B., Jala V.R. (2021). Regulation of intestinal barrier function by microbial metabolites. Cell. Mol. Gastroenterol. Hepatol..

[19] Chiang J.Y. (2013). Bile acid metabolism and signaling. Compr. Physiol..

[20] Wahlstrom A., Sayin S.I., Marschall H.U., Backhed F. (2016). Intestinal crosstalk between bile acids and microbiota and its impact on host metabolism. Cell Metab..

[21] Sinha S.R., Haileselassie Y., Nguyen L.P., Tropini C., Wang M., Becker L.S., Sim D., Jarr K., Spear E.T., Singh G. (2020). Dysbiosis-induced secondary bile acid deficiency promotes intestinal inflammation. Cell Host. Microbe.

[22] Hamilton J.P., Xie G., Raufman J.P., Hogan S., Griffin T.L., Packard C.A., Chatfield D.A., Hagey L.R., Steinbach J.H., Hofmann A.F. (2007). Human cecal bile acids: Concentration and spectrum. Am. J. Physiol. Gastrointest Liver Physiol..

[23] Wang K., Liao M., Zhou N., Bao L., Ma K., Zheng Z., Wang Y., Liu C., Wang W., Wang J. (2019). Parabacteroides distasonis alleviates obesity and metabolic dysfunctions via production of succinate and secondary bile acids. Cell Rep..

[24] Gerez J.R., Pinton P., Callu P., Grosjean F., Oswald I.P., Bracarense A.P.F.L. (2015). Deoxynivalenol alone or in combination with nivalenol and zearalenone induce systemic histological changes in pigs. Exp. Toxicol. Pathol..

[25] Payros D., Menard S., Laffitte J., Neves M., Tremblay-Franco M., Luo S., Fouche E., Snini S.P., Theodorou V., Pinton P. (2020). The food contaminant, deoxynivalenol, modulates the T helper/Treg balance and increases inflammatory bowel diseases. Arch. Toxicol..

[26] Pestka J.J., Smolinski A.T. (2005). Deoxynivalenol: Toxicology and potential effects on humans. J. Toxicol. Environ. Health B Crit. Rev..

[27] Pinton P., Oswald I.P. (2014). Effect of deoxynivalenol and other Type B trichothecenes on the intestine: A review. Toxins.

[28] Luu T.H., Bard J.M., Carbonnelle D., Chaillou C., Huvelin J.M., Bobin-Dubigeon C., Nazih H. (2018). Lithocholic bile acid inhibits lipogenesis and induces apoptosis in breast cancer cells. Cell. Oncol..

[29] Trah J., Arand J., Oh J., Pagerols-Raluy L., Trochimiuk M., Appl B., Heidelbach H., Vincent D., Saleem M.A., Reinshagen K. (2020). Lithocholic bile acid induces apoptosis in human nephroblastoma cells: A non-selective treatment option. Sci. Rep..

[30] Lajczak-McGinley N.K., Porru E., Fallon C.M., Smyth J., Curley C., McCarron P.A., Tambuwala M.M., Roda A., Keely S.J. (2020). The secondary bile acids, ursodeoxycholic acid and lithocholic acid, protect against intestinal inflammation by inhibition of epithelial apoptosis. Physiol. Rep..

[31] Song Y., Liu W., Zhao Y., Zang J., Gao H. (2021). Ochratoxin A induces human kidney tubular epithelial cell apoptosis through regulating lipid raft/PTEN/AKT signaling pathway. Environ. Toxicol..

[32] Song Y., Liu W., Zhao Y., Zang J., Gao H. (2021). Fumonisin B1 exposure induces apoptosis of human kidney tubular epithelial cells through regulating PTEN/PI3K/AKT signaling pathway via disrupting lipid raft formation. Toxicon.

[33] Riley R.T., Merrill A.H. (2019). Ceramide synthase inhibition by fumonisins: A perfect storm of perturbed sphingolipid metabolism, signaling, and disease. J. Lipid Res..

[34] Tardieu D., Matard-Mann M., Collen P.N., Guerre P. (2021). Strong alterations in the sphingolipid profile of chickens fed a dose of fumonisins considered safe. Toxins.

[35] Perino A., Demagny H., Velazquez-Villegas L., Schoonjans K. (2021). Molecular physiology of bile acid signaling in health, disease, and aging. Physiol. Rev..

[36] Cai D., Wang J., Jia Y., Liu H., Yuan M., Dong H., Zhao R. (2016). Gestational dietary betaine supplementation suppresses hepatic expression of lipogenic genes in neonatal piglets through epigenetic and glucocorticoid receptor-dependent mechanisms. Biochim. Biophys. Acta.

[37] Liu H.Y., Gu H., Qu H., Bao W., Li Y., Cai D. (2022). Aberrant cholesterol metabolic genes regulation in a negative feedback loop induced by an alphacoronavirus. Front. Nutr..

[38] Yousef I.M., Tuchweber B. (1984). Effect of lithocholic acid on cholesterol synthesis and transport in the rat liver. Biochim. Biophys. Acta..

[39] Cai D., Wang J., Gao B., Li J., Wu F., Zou J.X., Xu J., Jiang Y., Zou H., Huang Z. (2019). RORgamma is a targetable master regulator of cholesterol biosynthesis in a cancer subtype. Nat. Commun..

[40] Hang S., Paik D., Yao L., Kim E., Trinath J., Lu J., Ha S., Nelson B.N., Kelly S.P., Wu L. (2019). Bile acid metabolites control Th17 and Treg cell differentiation. Nature.

[41] Paik D., Yao L., Zhang Y., Bae S., D’Agostino G.D., Zhang M., Kim E., Franzosa E.A., Avila-Pacheco J., Bisanz J.E. (2022). Human gut bacteria produce TauEta17-modulating bile acid metabolites. Nature.

[42] Chiang J.Y.L., Ferrell J.M. (2018). Bile acid metabolism in liver pathobiology. Gene Expr..

[43] Lunney J.K., Van Goor A., Walker K.E., Hailstock T., Franklin J., Dai C.H. (2021). Importance of the pig as a human biomedical model. Sci. Transl. Med..

